# Comparable joint awareness and implant survival at midterm follow‐up between CR and PS TKA: An anatomic phenotype‐based propensity score‐matched analysis

**DOI:** 10.1002/jeo2.70242

**Published:** 2025-05-19

**Authors:** Lars‐Rene Tuecking, Tobias Welzel, Max Ettinger, Henning Windhagen, Peter Savov

**Affiliations:** ^1^ Department of Orthopaedic Surgery Hannover Medical School, Diakovere Annastift Hannover Germany; ^2^ Department of Orthopaedic and Trauma Surgery, Pius Hospital Carl von Ossietzky University of Oldenburg Oldenburg Germany

**Keywords:** CR, implant survival, joint awareness, PS, total knee arthroplasty

## Abstract

**Purpose:**

Recent studies highlight the role of joint awareness and the influence of preoperative anatomy on outcomes of cruciate‐retaining (CR) versus posterior‐stabilized (PS) implants in total knee arthroplasty (TKA). There is currently a lack of studies comparing CR and PS prostheses while adjusting for important anatomical parameters and anatomical phenotypes with large group sizes.

**Methods:**

This retrospective single‐centre study analyzed patients who underwent primary TKA with the Triathlon® CR or PS implant system from 2008 to 2014 with a minimum follow‐up of 6.5 years. Patients were matched using propensity scores based on demographics (age, gender and body mass index) and preoperative anatomic angle parameters (lateral distal femoral angle [LDFA], medial proximal tibia angle, hip–knee–ankle angle [HKA], arithmetic HKA and joint line obliquity) and Coronal Plane Alignment of the Knee (CPAK) types. Outcome data included patient‐reported outcomes (PROMs: Forgotten Joint Score, Oxford Knee Score, Knee Society Score, Western Ontario and McMaster Universities Osteoarthritis Index, visual analogue scale and University of California at Los Angeles), demographic data, post‐operative clinical course data. Statistical analysis was conducted using R, with significance set at *p* < 0.05.

**Results:**

A total of 728 patients (513 CR and 215 PS) were included, leaving 519 patients (346 CR and 173 PS) being analyzed after propensity score matching. Joint awareness and further clinical scores showed no differences between CR and PS implants (*p* > 0.05). Implant survival at 5 and 10 years was similar for both types (log‐rank test: *p* = 0.164 and *p* = 0.163), though CR implants had lower survival rates overall. Valgus CPAK types III and VI showed the lowest survival rates, especially for CR implants. Regression analysis revealed younger patient age significantly affected CR implant survival, while increasing valgus LDFA decreased PS implant survival.

**Conclusion:**

No differences were found in the joint awareness of CR and PS prostheses in the medium to long‐term follow‐up, while controlling for preoperative anatomy. Similarly, there were no significant variations in implant survival. Noticeably higher revision rates in the valgus CPAK phenotypes were found for both systems. A high valgus LDFA angle was identified as a risk factor for revisions in PS systems.

**Level of Evidence:**

Level III.

**Abbreviations**
aHKA: arithmetic hip–knee–ankle angle
BMI: body mass index
CPAK: Coronal Plane Alignment of the Knee
CR: cruciate retaining
FJS: Forgotten Joint Score
HKA: hip–knee–ankle angle
JLO: joint line obliquity
LDFA: lateral distal femoral angle
MA: mechanical alignment
MPTA: medial proximal tibial angle
PROM: patient‐reported outcome measure
PS: posterior stabilized
TKA: total knee arthroplasty
WOMAC: Western Ontario and McMaster Universities Osteoarthritis Index

## INTRODUCTION

The comparison between cruciate‐retaining (CR) and posterior‐stabilized (PS) total knee arthroplasty (TKA) has been extensively studied through various studies and meta‐analyses [14, 20], mostly showing no significant differences in knee scores, radiological outcomes, and complications between CR and PS TKA [14]. While PS TKA tends to show improved maximum knee flexion [18], the current national implant registry tends to show reduced implant survival for these implant systems compared to CR TKA [27, 31]. For this reason, the decision as to whether CR or PS TKA is still primarily based on the surgeon's preference and experience, if no strict contraindication for PCL retention appears. The influence of different operative techniques, computer‐ and robotic‐assistance and in particular prosthesis types on joint awareness has recently been investigated in individual studies [9, 15, 16, 17, 26, 33, 34, 36, 37]. In a small case series comparing CR and PS TKA, the authors found reduced joint awareness in CR TKA [34]. A possible reason for this was considered to be an improvement in proprioception due to the preservation of the PCL in CR TKA [39], although other studies have already contradicted this hypothesis [1, 5]. Furthermore, medium‐ to long‐term studies evaluating joint awareness between CR and PS TKA are important, as secondary degeneration of the PCL may occur in the long‐term course after CR TKA [24, 40]. This has a significant influence on the stability of the TKA and, accordingly, on clinical outcomes and implant survival. In this context, Broberg et al. showed that the long‐term functional outcome in PS TKA is superior to CR TKA [6]. In addition to the type of prosthesis, the arthritic anatomy also appears to have an influence on the results of CR and PS TKA. Despite the absence of clinical differences between CR and PS TKA in high‐grade varus patients [30], particularly a reduction in clinical outcome and implant survival was observed in valgus patients [28]. Since gross differentiated anatomies of the leg axis and the knee joint (varus vs. valgus) already have an influence on the outcome of PS versus CR TKA, the question of the influence of different knee phenotypes on the outcome of PS versus CR TKA arises. In addition, studies comparing the clinical outcome of CR and PS TKA should make the best possible adjustment for the preoperative anatomy in order to be able to carry out a comparison that is as bias‐free as possible. In addition, these studies should include joint awareness scores in particular. There are currently few studies on the structured comparison of CR and PS prostheses with regard to joint awareness. Many older studies used other PROM scores for comparison, some of which poorly reflect the correct satisfaction of the patient [10] and some of which have a high ceiling effect, thus limiting comparability, particularly in the case of good and very good results [7]. Scores such as the Forgotten Joint Score (FJS), on the other hand, can also correctly reflect the satisfaction and joint awareness of patients in a longitudinal comparison [19].

For these reasons, the aim of this study was to perform comparison between CR and PS TKA with a propensity‐score control for the preoperative anatomy of the limb alignment comparing implant survival and clinical outcome scores with focus on joint awareness using the FJS.

## METHODS

### Study design

This is a single‐centre retrospective study conducted on patients who underwent primary TKA from 2008 to 2014 with a single implant system (Triathlon®, Stryker) either using CR or PS constraint level at tertiary arthroplasty centre. Patients were assigned to one of two groups (CR or PS) based on the degree of prosthetic constraint. To ensure the minimalization of selection bias, a propensity score‐matched analysis of the outcome measures based on patient demographics, preoperative anatomy and knee phenotype was conducted. All data was collected retrospectively via a self‐administered patient‐reported outcome measurement (PROM) survey. Ethical approval for this study was obtained from the local ethics committee (Nr. 8403_BO_S_2019). Written informed consent was obtained from every participating patient.

### Inclusion and exclusion criteria

All patients operated with the Triathlon® CR or PS system from 2008 to 2014 undergoing primary TKA surgery for primary osteoarthritis of the knee were included. Exclusion criteria were different implant systems, revision TKA procedures (e.g., UKA revision to PS system), secondary arthritis (posttraumatic, rheumatoid) and mobility impairment due to secondary diagnoses (e.g. neurological pathologies).

### Operative technique

All operations were conducted in a single institution by senior surgeons with >50 TKA/year. Operative technique and implant selection were mainly dependent on individual surgeon's personal preferences. Rare contraindications for CR prostheses, such as posterior cruciate ligament insufficiency or severe extension deficits exceeding 20° without posterior osteophytes, were considered. All patients received a cemented Triathlon® CR or PS implant (fixed bearing) via a medial parapatellar approach. A posterior‐referenced measured‐resection technique aiming for mechanical alignment (MA) was performed in all cases. The soft tissue releases were performed in a sequential manner, starting with the deep medial collateral ligament (MCL), followed by the superficial MCL, and then the posteromedial capsule as needed, to ensure sufficient medial release and knee stability. In valgus cases, a stepwise approach was standard at this surgery centre. The following structures were released stepwise depending on the severity of valgus deformity and intraoperative soft tissue balance (first to last): iliotibial band, lateral retinaculum, popliteus tendon, posterolateral capsule and lateral collateral ligament.

### Outcome measures and data collection

**Figure 1:**
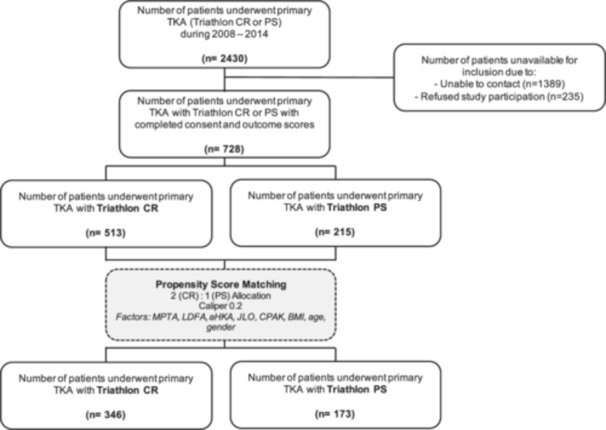
Group allocation of patients. Flow chart of group allocation and matching procedure. aHKA, arithmetic hip–knee–ankle angle; BMI, body mass index; CPAK, Coronal Plane Alignment of the Knee; CR, cruciate retaining; JLO, joint line obliquity; LDFA, lateral distal femoral angle; MPTA, medial proximal tibial angle; PS, posterior stabilized.

Demographic data was extracted from the institutional clinical information system. All patients operated from 2008 to 2014 with the above‐mentioned TKA system were recorded. Subsequently, patients were contacted by mail, and written informed consent was obtained. All patient‐reported outcome measures (PROMs) described below were collected via self‐administered questionnaires, and a survey on implant survival was conducted. All patients without a postal response were contacted by telephone and reminded of their possible participation. Patients who could not be contacted by post or telephone, or with missing and rejected informed consent were finally excluded (see Figure 1). PROMs for post‐operative clinical outcome included FJS, Oxford Knee Score, Knee Society Score, Western Ontario and McMaster Universities Osteoarthritis Index (WOMAC), visual analogue scale (VAS) and University of California at Los Angeles activity‐level scale. Demographic data at the time of TKA surgery included gender, body mass index (BMI) and implant specifications (CR/PS, size, onlay height in mm, with/without patellar resurfacing). The radiological examination included long knee radiographs in two planes with additional full‐leg radiographs preoperatively. Radiological measurements included hip‐to‐knee‐ankle angle (HKA), lateral distal femoral angle (LDFA), medial proximal tibia angle (MPTA), arithmetic HKA (aHKA = MPTA − LDFA) and joint line obliquity (JLO = MPTA + LDFA). Using aHKA and JLO, the Coronal Plane Alignment of the Knee (CPAK) type was calculated for each patient [22].

### Propensity score matching (PSM)

In addition to demographic factors such as age [35], gender [41] and BMI [4], preoperative anatomical factors also influence the outcome after TKA surgery. Especially with regard to the comparison of CR and PS implants (e.g., due to differences in rotational stability in flexion), preoperative anatomical variables and phenotypes might be possible factors influencing clinical outcomes after TKA. Accordingly, age, gender, BMI, MPTA, LDFA, HKA, aHKA, JLO and CPAK type were defined as confounding factors. Due to the large available number of CR patients in this study, a 2:1 (CR:PS) allocation was performed. A calliper of 0.2 was used for propensity score calculation. Two PSM analyses were carried out, one for the survival analysis and the other for the comparison of the clinical scores of revision‐free patients. All revised patients were excluded for the comparison of the PROMs, as a revision or implant replacement naturally has a significant influence on the clinical outcome, and the clinical result of the primary implantation can no longer be analyzed in these cases.

### Statistical analysis

Statistical analysis was conducted in R (R version 4.3.2, R Core Team, 2023). Categorical data of groups was compared using Pearson chi‐square tests; continuous data were compared using independent *t* tests after controlling for normal distribution with the Shapiro–Wilk test. Continuous data were described with mean ± standard deviation, whereas categorical data were described as numbers and frequencies. Clinical outcome scores of both groups were sorely compared between revision‐free patients (CR: 312 and PS: 165). Survival analysis was conducted using the Kaplan–Meier method (revision defined as event). Patients were censored at the date of death. Survival curves were compared using the log‐rank (Mantel–Cox) test. Cox regression analysis was used to test for factors that influence implant survival. A *p* value < 0.05 was defined as statistical significance.

## RESULTS

### Study cohort and demographic data after PSM

A total of 728 patients (CR: 513 and PS: 215) were included with full PROM data and written informed consent (Figure 1, Table 1). After PSM with a 2:1 (CR:PS) allocation and caliper of 0.2, a total of 519 patients were included. Of these, 346 patients were treated with a CR TKA, whereas 173 patients were treated with a PS TKA. After PSM, demographic data between groups was comparable with low standardized mean differences (<0.1) and no significant differences in the main demographic parameters (Table 2).

**Table 1:** Demographic data before propensity score matching.

	Cruciate retaining	Posterior stabilized	*p* value	SMD
Group size	513	215		
Age (years)	65.79 (±8.80)	66.85 (±9.17)	0.143	0.118
Sex (female)	326 (63.5%)	136 (63.3%)	1.000	0.006
BMI (kg/m^2^)	30.38 (±5.26)	30.11 (±5.26)	0.605	0.051
HKA (°)	176.5 (±6.44)	179.1 (±8.60)	**<0.001**	0.335
MPTA (°)	87.65 (±3.00)	87.90 (±4.10)	0.368	0.070
LDFA (°)	88.29 (±2.81)	87.20 (±3.72)	**<0.001**	0.332
aHKA (°)	−0.64 (±4.52)	0.70 (±6.28)	**0.002**	0.246
JLO (°)	175.94 (±3.65)	175.10 (±4.67)	**0.011**	0.201
CPAK				
Type 1	122 (24.5%)	59 (28.8%)	NA	0.528
Type 2	109 (21.9%)	29 (14.1%)		
Type 3	76 (15.3%)	64 (31.2%)		
Type 4	58 (11.6%)	11 (5.4%)		
Type 5	70 (14.1%)	14 (6.8%)		
Type 6	46 (9.2%)	19 (9.3%)		
Type 7	6 (1.2%)	3 (1.5%)		
Type 8	4 (0.8%)	1 (0.5%)		
Type 9	6 (1.2%)	5 (2.4%)		
Onlay height (mm)	10.05 (±1.51)	10.92 (±2.46)	**<0.001**	0.423
Patella resurfacing				
No	406 (79.1%)	155 (72.1%)	**0.031**	0.237
Yes	107 (20.9%)	77 (27.9%)		

*Note*: Mean ± standard deviation. Bold values indicate statistically significant.

Abbreviations: aHKA, arithmetic HKA; BMI, body mass index; CPAK, Coronal Plane Alignment of the Knee; HKA, hip–knee–ankle angle; JLO, joint line obliquity; LDFA, lateral distal femoral angle; MPTA, medial proximal tibia angle; SMD, standardized mean difference.

**Table 2:** Demographic data after propensity score matching.

	Cruciate retaining	Posterior stabilized	*p* value	SMD
Group size	346	173		
Age (years)	65.65 (±9.14)	67.17 (±8.97)	0.073	0.096
Sex (female)	222 (64.2%)	108 (62.4%)	0.700	0.036
BMI (kg/m^2^)	30.61 (±5.19)	30.01 (±4.87)	0.296	0.098
HKA (°)	176.91 (±7.09)	177.76 (±7.79)	0.215	0.094
MPTA (°)	87.64 (±3.21)	87.54 (±4.16)	0.767	0.026
LDFA (°)	88.05 (±2.84)	87.98 (±3.03)	0.792	0.024
aHKA (°)	−0.41 (±4.86)	−0.44 (±5.74)	0.956	0.005
JLO (°)	175.70 (±3.61)	175.53 (±4.47)	0.643	0.042
CPAK				
Type 1	113 (32.7%)	57 (32.9%)	NA	0.062
Type 2	55 (15.9%)	29 (16.8%)		
Type 3	75 (21.7%)	36 (20.8%)		
Type 4	25 (7.2%)	11 (6.4%)		
Type 5	28 (8.1%)	14 (8.1%)		
Type 6	35 (10.1%)	19 (11.0%)		
Type 7	6 (1.7%)	3 (1.7%)		
Type 8	3 (0.9%)	1 (0.6%)		
Type 9	6 (1.7%)	3 (1.7%)		
Onlay height (mm)	10.05 (±1.56)	10.23 (±2.07)	0.253	0.101
Patella resurfacing				
No	274 (79.2%)	128 (74.0%)	0.181	0.058
Yes	72 (20.8%)	45 (26.0%)		

*Note*: Mean ± standard deviation.

Abbreviations: aHKA, arithmetic hip–knee–ankle angle; BMI, body mass index; CPAK, Coronal Plane Alignment of the Knee; JLO, joint line obliquity; LDFA, lateral distal femoral angle; MPTA, medial proximal tibia angle; SMD, standardized mean difference.

### Clinical outcome parameters

The results of the clinical outcome scores are presented in Table 3. No statistically significant difference was observed in joint awareness between the CR and PS prostheses at the medium‐ to long‐term follow‐up (CR: 59.4 vs. PS: 60.2, *p* = 0.79). All other clinical scores also showed no statistically significant differences between the two groups.

**Table 3:** Clinical outcome scores.

	Cruciate retaining	Posterior stabilized	*p* value
Forgotten Joint Score	59.41 (±29.06)	60.22 (±29.66)	0.793
Oxford Knee Score	25.04 (±10.37)	25.69 (±10.76)	0.563
WOMAC Score			
Total	15.45 (±18.42)	15.73 (±19.76)	0.900
Pain	12.62 (±16.68)	13.96 (±20.07)	0.483
Stiffness	14.33 (±18.73)	18.13 (±23.41)	0.105
Function	16.71 (±20.09)	17.08 (±20.93)	0.867
Knee Society Score			
Expectation	10.26 (±3.05)	10.68 (±3.32)	0.217
Satisfaction	31.63 (±8.33)	31.38 (±9.37)	0.788
Function	72.19 (±21.77)	72.46 (±23.38)	0.913
UCLA activity score	4.79 (±1.62)	5.11 (±1.64)	0.061
VAS score			
Rest	0.74 (±1.48)	0.93 (±1.85)	0.272
Activity	1.84 (±2.41)	1.84 (±2.44)	0.996

*Note*: Mean ± standard deviation.

Abbreviations: UCLA, University of California at Los Angeles; VAS, visual analogue scale; WOMAC, Western Ontario and McMaster Universities Osteoarthritis Index.

### Implant survival analysis

**Figure 2:**
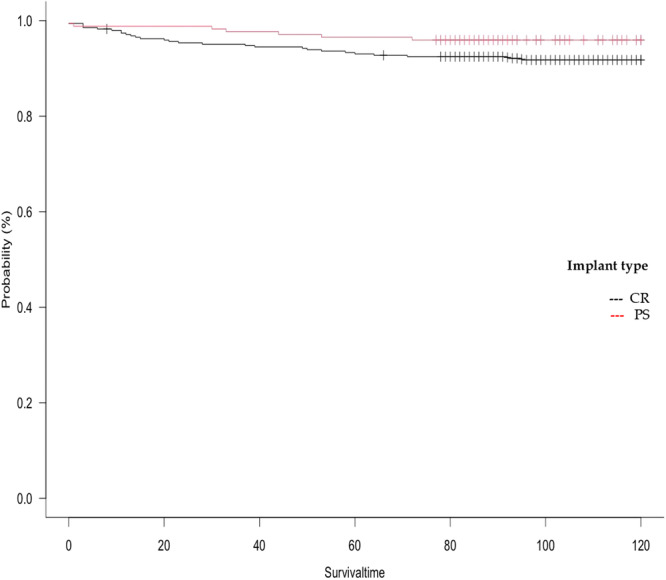
Kaplan–Meier curve: Total implant survival. Total implant survival comparing CR and PS implant types. Survival time in months. CR, cruciate retaining; PS, posterior stabilized.

**Figure 3:**
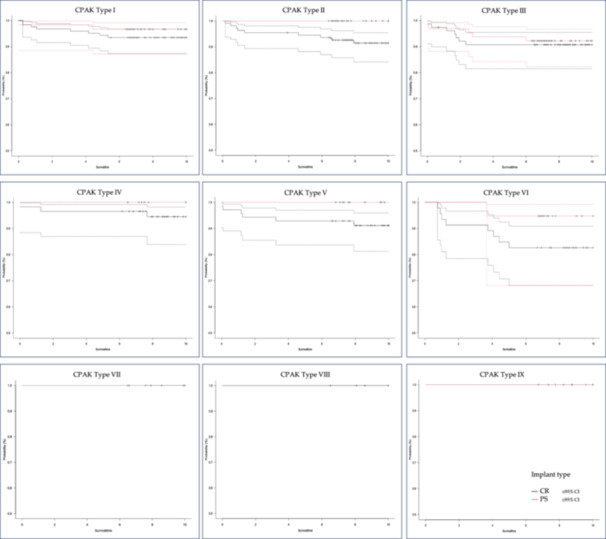
Kaplan–Meier curve: CPAK‐based implant survival. Implant survival comparing CR and PS implant types in different CPAK types. Survival time in years, survival probability beginning with 50%. CPAK, Coronal Plane Alignment of the Knee; CR, cruciate retaining; PS, posterior stabilized.

As shown in Table 4, the analysis of implant survival demonstrated no statistically significant difference between CR and PS prostheses at five and ten years post‐operatively (Figure 2, log‐rank test: *p* = 0.164 and *p* = 0.163, respectively). However, the survival rate at both time points was lower for the CR prosthesis compared to the PS prosthesis. With regard to the preoperative CPAK types (Figure 3), the lowest survival rates for CR and PS prostheses were observed in valgus CPAK types III and VI. The lowest survival rate in these cohorts was observed in CPAK type VI patients who were treated with CR prostheses (82.9%). The main revision causes for CR implants were instability (27.6%), aseptic loosening (17.2%) and stiffness (17.2%), whereas causes for PS implant revisions were mainly aseptic loosening (50.0%), stiffness (12.5%) and instability (12.5%).

**Table 4:** Survival analysis with subgroup CPAK survival analysis.

	Cruciate retaining	Posterior stabilized	*p* value
Total			
5‐Year survival	**93.0%**	**96.5%**	**0.164**
10‐Year survival	**91.8%**	**96.0%**	**0.163**
CPAK			0.389
Type 1	**92.9%**	**96.5%**	
Type 2	94.5%	100.0%	
Type 3	**90.6%**	**88.9%**	
Type 4	100.0%	100.0%	
Type 5	92.9%	100.0%	
Type 6	**82.9%**	**94.7%**	
Type 7	100.0%	100.0%	
Type 8	100.0%	100.0%	
Type 9	100.0%	100.0%	

*Note*: Mean ± standard deviation. Bold values indicate statistically significant.

Abbreviation: CPAK, Coronal Plane Alignment of the Knee.

### Influencing factors on implant survival

The results of regression analysis are shown in Table 5. Demographic factors only showed a significant influence in CR‐type implants, with a decreased risk of revision of 6%/year of age. Anatomic factors showed no influence on implant survival in CR‐type implants, whereas the preoperative LDFA showed a significant influence on survival of PS‐type implants with a risk reduction of 21% with each increasing LDFA degree (increasing varus LDFA). Further implant‐specific factors (e.g., the presence of patellar resurfacing) showed no significant influence on implant survival.

**Table 5:** Cox regression analysis on implant survival influencing factors.

	Cruciate retaining				Posterior stabilized			
	Hazard ratio	Lower 95% CI	Upper 95% CI	*p* value	Hazard ratio	Lower 95% CI	Upper 95% CI	*p* value
Age	**0.9459**	0.9106	0.9826	**0.0042**	0.9874	0.9137	1.067	0.7486
BMI	1.063	0.9844	1.149	0.1184	1.087	0.8898	1.329	0.4127
aHKA	1.023	0.9494	1.103	0.5454	1.086	0.9576	1.232	0.1987
JLO	0.9801	0.885	1.085	0.7003	0.9244	0.7994	1.069	0.2892
LDFA	0.9511	0.8367	1.081	0.4436	**0.7867**	0.6211	0.9965	**0.04669**
MPTA	1.014	0.9053	1.136	0.8108	1.026	0.8741	1.205	0.7528
Onlay height	0.8214	0.6056	1.114	0.2058	0.909	0.698	1.184	0.4791
Patellar resurfacing	1.434	0.6347	3.24	0.3861	1.545	0.756	4.31	0.3745

*Note*: Bold values indicate statistically significant.

Abbreviations: aHKA, arithmetic hip–knee–ankle angle; BMI, body mass index; CI, confidence interval; CPAK, Coronal Plane Alignment of the Knee, JLO, joint line obliquity, LDFA, lateral distal femoral angle; MPTA, medial proximal tibia angle.

## DISCUSSION

The main result of this study was that despite matching the preoperative anatomy and phenotypes of the knee joint, no differences in joint awareness were found between CR and PS prostheses in the medium‐ to long‐term follow‐up. In contrast to individual studies that showed reduced joint awareness after CR implants [34], no differences in joint awareness were found in the current study when matching the preoperative anatomy. The hypothesis that retention of the PCL may improve joint awareness by possibly improving proprioception could not be confirmed in our study. Further studies showed an improved functional outcome of PS prostheses in the long‐term follow‐up, which may be associated with secondary degeneration of the PCL and loss of femoral rollback in CR implants [6]. We were also unable to show this after matching the preoperative anatomy in the medium‐ and long‐term follow‐ups for the comparison of CR and PS prostheses. Similar results have already been shown in numerous meta‐analyses comparing CR and PS prostheses with no differences in clinical outcome in either the short‐ or long‐term follow‐up [5, 14, 18]. Nevertheless, no study with medium‐ and long‐term follow‐up has so far controlled for the preoperative anatomy and preoperative phenotypes of the knee joint with a comparable total case number, to the authors' knowledge. For this reason, this study seems to be able to exclude the possibility that the clinical outcome in the direct comparison of CR and PS implants is influenced by preoperative anatomical factors and phenotypes. In this study, in addition to the phenotypes, the individual anatomical parameters were also used as the basis for propensity matching. This is important to note, as the CPAK classification is only a simplification of a few groups and only considers the coronal level, ignoring the sagittal level. Other classifications, such as the Functional Knee Phenotype Classification by Hirschmann et al. [13], describe the variation in knee anatomy much more precisely and would possibly provide even more information about anatomical influences on the outcome of CR and PS prostheses. Unfortunately, the present study size was not sufficient to perform clustered statistical analyses based on the preoperative Functional Knee Phenotype.

In addition to evaluating the clinical outcome, this study also assessed implant survival. The primary finding of this study was that no significant differences were observed in implant survival rates after 5 and 10 years, although the CR implants displayed a slightly higher revision rate. This partly contradicts the registry data from Germany, Australia, and the United Kingdom, which indicates lower revision rates for CR implants compared to PS implants [8, 29, 32]. Nevertheless, single‐centre studies of highly specialized centres often show divergent revision rates or clinical outcome results when compared directly to large registry data [2, 3]. One possible explanation for these differences is the isolated accumulation of the investigated procedure in a single centre, leading to reduced revision rates compared to the average registry data for that specific procedure [2, 3]. Nonetheless, a recent review comparing another prosthesis system (Attune TKA system, DePuy Synthes) also found no differences between CR and PS prostheses in terms of implant survival in the short term [38]. Additionally, the sub‐analysis of the CPAK types in both implant systems was notable with regard to revisions. The valgus phenotypes demonstrated higher revision rates for both implant systems, with CPAK III and particularly CPAK VI types exhibiting higher revision rates for CR prostheses compared to PS protheses. This is in agreement with other individual studies reporting increased revision rates for valgus patients [23], especially those receiving CR prostheses [28]. On the other hand, cox regression analysis also indicated a substantially increased risk of revision for patients with progressively valgus preoperative LDFA angles, which is evident in the PS prostheses in our cohort. One possible explanation could be that a pronounced correction of the femoral valgus in MA can lead to an elevation of the medial joint line, which can lead to mid‐flexion instability with increased proximalization of the joint line [21]. Considering increased medial laxity in valgus knees, this might lead to a paradoxical anterior shift in the medial compartment in early flexion [25]. All this, combined with low anteroposterior stability in the initial flexion phase of PS prostheses and increased rotational laxity in initial flexion in these implant types [11], might lead to overloading of the ventral and medioventral (e.g., medial retinaculum) structures, persistent instability and increasing risk of revision.

Several limitations need to be acknowledged in this research. The study's primary limitation arises from its retrospective design, which inevitably impacts the quality of the collected data and the resulting conclusions. Due to the follow‐up period of up to 10 years, a large proportion of the identified patients could not be reached during the retrospective data collection. Accordingly, further revisions or patients with limited clinical outcomes could possibly not be included. However, this limitation likely affected both groups to the same extent. Furthermore, propensity matching is mainly based on the preoperative anatomy. It was not possible to control for post‐operative alignment in this retrospective cohort, as post‐operative full‐leg radiographs were not yet standard at that time. Nevertheless, the operations were performed by a small number of senior surgeons with the same surgical philosophy and the same implant system in a single centre, and the group size should at least compensate for minor deviations in this manner. Matching was based on coronal anatomical parameters and the CPAK classification. Further analysis with other classification systems, such as the Functional Knee Phenotype Classification or a more recent classification into normal, deviant and aberrant coronal anatomies of the knee, could possibly allow better group‐specific analyses [12]. Nonetheless, numerous anatomical factors were already used as the basis for matching; additional parameters would have significantly restricted propensity matching and reduced group size even further.

## CONCLUSION

In conclusion, no differences were found in the joint awareness of CR and PS prostheses in the medium‐ to long‐term follow‐up, while controlling for preoperative anatomy. Similarly, there were no significant variations in implant survival; however, there were noticeably higher revision rates in the valgus CPAK phenotypes for both systems. A high valgus LDFA angle was identified as a risk factor for revisions in PS systems.

## AUTHOR CONTRIBUTIONS

**Lars‐Rene Tuecking**: Conceptualization; formal analysis; investigation; methodology; project administration; visualization; writing—original draft; writing—review and editing. **Max Ettinger**: Conceptualization; methodology; project administration; supervision; writing—review and editing. **Peter Savov**: Conceptualization; formal analysis; methodology; project administration; visualization; writing—original draft; writing—review and editing. **Tobias Welzel**: Data curation; investigation; visualization; writing—original draft. **Henning Windhagen**: Methodology; project administration; supervision; writing—review and editing.

## CONFLICT OF INTEREST STATEMENT

Max Ettinger, Lars‐Rene Tuecking, Peter Savov and Henning Windhagen have been paid educational consultancy fees from Stryker, USA. The remaining author declares no conflicts of interest.

## ETHICS STATEMENT

This study was approved by the local Ethics Committee (EK‐Nr. 8403_BO_S_2019). Written informed consent was received from every included patient.

## Data Availability

The data that support the findings of this study are available from the corresponding author upon reasonable request.
